# Intra- and peri-tumoral radiomics for predicting the sentinel lymph node metastasis in breast cancer based on preoperative mammography and MRI

**DOI:** 10.3389/fonc.2022.1047572

**Published:** 2022-12-12

**Authors:** Yuan Cheng, Shu Xu, Haotian Wang, Xiaoyu Wang, Shuxian Niu, Yahong Luo, Nannan Zhao

**Affiliations:** ^1^ Department of Biomedical Engineering, School of Intelligent Medicine, China Medical University, Shenyang, China; ^2^ Department of Radiology, Cancer Hospital of China Medical University, Liaoning Cancer Hospital and Institute, Shenyang, China

**Keywords:** breast cancer, sentinel lymph node metastasis, mammography, MRI, radiomics

## Abstract

**Purpose:**

This study aims to investigate values of intra- and peri-tumoral regions in the mammography and magnetic resonance imaging (MRI) image for prediction of sentinel lymph node metastasis (SLNM) in invasive breast cancer (BC).

**Methods:**

This study included 208 patients with invasive BC between Spe. 2017 and Apr. 2021. All patients underwent preoperative digital mammography (DM), digital breast tomosynthesis (DBT), dynamic contrast-enhanced MRI (DCE-MRI) and diffusion-weighted MRI (DWI) scans. Radiomics features were extracted from manually outlined intratumoral regions, and automatically dilated peritumoral tumor regions in each modality. The least absolute shrinkage and selection operator (LASSO) regression was used to select key features from each region to develop radiomics signatures (RSs). Area under the receiver operating characteristic curve (AUC), accuracy, sensitivity, specificity and negative predictive value (NPV) were calculated to evaluate performance of the RSs.

**Results:**

Intra- and peri-tumoral regions of BC can provide complementary information on the SLN status. In each modality, the Com-RSs derived from combined intra- and peri-tumoral regions always yielded higher AUCs than the Intra-RSs or Peri-RSs. A total of 10 and 11 features were identified as the most important predictors from mammography (DM plus DBT) and MRI (DCE-MRI plus DWI), respectively. The DCE-MRI plus DWI generated higher AUCs compared with DM plus DBT in the training (AUCs, DCE-MRI plus DWI vs. DM plus DBT, 0.897 vs. 0.846) and validation (AUCs, DCE-MRI plus DWI vs. DM plus DBT, 0.826 vs. 0.786) cohort.

**Conclusions:**

Radiomics features from intra- and peri-tumoral regions can provide complementary information to identify the SLNM in both mammography and MRI. The DCE-MRI plus DWI generated lower specificity, but higher AUC, accuracy, sensitivity and negative predictive value compared with DM plus DBT.

## Introduction

Breast cancer (BC) has been the most frequently diagnosed malignant disease in females and the second leading cause of cancer mortality amongst women worldwide (1). The sentinel lymph node (SLN) is the first draining site that can be affected when cancer cells spread from the primary breast tumor (2), and hence the SLN status is a crucial parameter for staging, treatment planning and prognosis (3, 4). Postoperative chemoradiotherapy is needed once the lymph node metastasis is histopathologically detected after surgery, which often cause adverse effects and would be avoided. In clinical, the sentinel lymph node biopsy (SLNB) is routinely used as a standard procedure to evaluate the SLN status, but may cause potential significant complications due to the invasive operation (5). Besides, the SLNB relies on experiences of the operators with unstandardized radiopharmaceuticals, and may lead to a potentially high false-negative rate, which hinders its clinical efficiency (6, 7). Thus, there is a great need for developing an accurate and noninvasive technique to preoperatively evaluate the SLM status.

Mammography and Magnetic Resonance Imaging (MRI) screenings are commonly used in the diagnosis, staging and prognosis of BC (8). The MRI screening is sensitive, but has disadvantages e.g. the specificity and high examination fees, and is not suitable for all patients (9). While, the mammography techniques, including full-field digital mammography (FFDM) and digital breast tomosynthesis (DBT) are widely used in clinical as routine screening methods, and accessible to all patients. However, their capabilities for evaluating the lymph node metastasis by visual inspection are limited, since there is still no specific biomarker (10).

The radiomics has been increasingly utilized to capture valuable markers in BC for the diagnosis, prediction of gene expression and prognosis (11), because the tumor characteristics can be comprehensively assessed from the whole tumor region in the medical image (12), rather than from limited biopsy tissue samples. Many radiomics studies have been conducted on the diagnosis, therapeutic response prediction and prognosis in BC (13). Previous reports also revealed associations between the lymph node status and MRI-based radiomics features (2, 14), but both focused on the intratumoral tumor region, without considering information from peritumoral regions. While, increasing evidences have demonstrated that peritumoral regions may hold great information associated with tumor characteristics (15–17). A recent effort also suggested that the peritumoral region of BC in the MRI image holds valuable information regarding the SLN status (18). While, the study only evaluated DCE-MRI and provided numerical results (e.g., AUC), which hindered the clinical value. To our knowledge, no previous report has been released on quantitatively evaluating and comparing prediction capabilities of mammography and MRI individually and in combination on the SLN status based on intra- and peri-tumoral regions of BC.

## Materials and methods

### Patients

**Figure 1:**
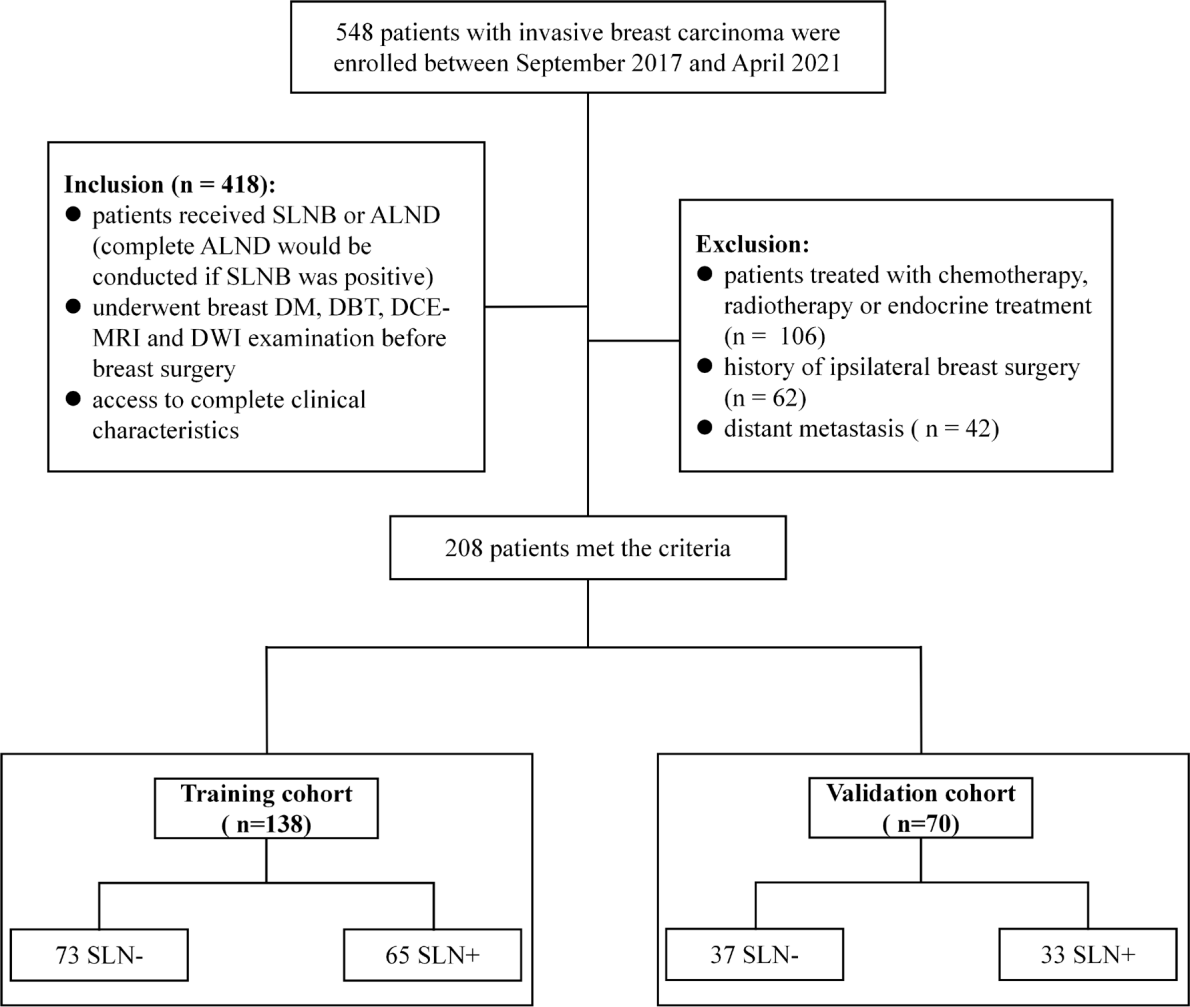
Patient recruitment process in this study. SLN-, sentinel lymph node negative; SLN+, sentinel lymph node positive.

The retrospective study was approved by the Institutional Review Board of our hospital. A total of 548 patients histopathologically confirmed invasive breast carcinoma were consecutively enrolled between Sep. 2017 and Apr. 2021. Inclusion criteria were: 1) patients received SLNB or ALND (complete ALND would be conducted if the SLN was positive); 2) underwent breast DM, DBT, DCE-MRI and DWI examination before breast surgery; and 3) access to complete clinical characteristics. Exclusion criteria were: 1) patients treated with chemotherapy, radiotherapy or endocrine treatment; 2) history of ipsilateral breast surgery; and 3) distant metastasis. Clinical characteristics were collected from patients’ medical records, including age, menstruation status, tumor location, histological grade, histological type, estrogen receptor (ER) status, progesterone receptor (PR) status, human epidermal growth factor receptor-2 (HER-2) status and Ki-67 level. Mann-Whitney U test and Chi-Square test were applied on clinical characteristics to identify the most important clinical predictors. A P<0.05 was considered significant. A total of 208 patients who met the criteria were finally included and randomized into the training (n = 138) and the validation cohort (n = 70) at a 2:1 ratio. **Figure 1** showed a flow chat of the patient recruitment.

### Imaging acquisition and tumor segmentation

The DM and DBT screenings were performed using a Hologic scanner (Hologic Selenia Dimensions, HOLGIC, USA). The mammography positions were standard craniocaudal (CC) position and mediolateral oblique (MLO) position. For the DCE-MRI and DWI screening, a 1.5 T MRI scanner (HDx, GE Healthcare) with an eight channels array was used. Mammography and MRI imaging parameters were as follows: (i) The voltage range on the X-ray tubes = 20.0-49.0 kv (step = 1.0 kv), current time range = 300-400 mAs, nominal power = 3.0 kW, scanning time < 4.0 s, reconstruction time = 2.0-5.0 s, and pixel size = 70 μm. (ii) The DCE-MRI scanning was performed with the repetition time (TR) = 6.2 ms, echo time (TE) = 3.0 ms, field of view (FOV) = 360 × 360 mm, matrix size = 256 × 256, layer thickness = 3.2 mm, flip angle = 10 degree, 48 slices per volume. (iii) The DWI scanning was performed with TR = 6000 ms, TE = 64 ms, FOV = 350 × 350 mm, matrix size = 128 × 128, layer thickness = 6 mm, flip angle = 90 degrees, b-value = 800 s/mm².

**Figure 2:**
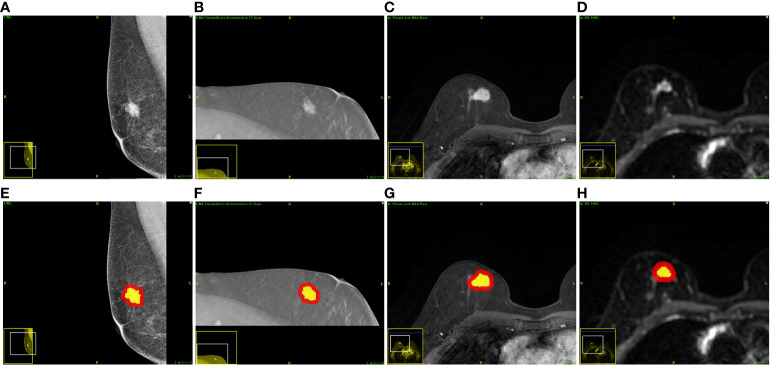
Examples of one patient who had invasive breast cancer and the segmented ROIs. From left to right: **(A, E)** the DM image, **(B, F)** the DBT image, **(C, G)** the DCE-MRI image, and **(D, H)** the DWI image. The yellow regions are the intratumoral ROIs. The red regions indicate the peritumoral ROIs.

For each modality, the intratumoral region of interests (ROIs) were manually delineated slice by slice using the ITK-SNAP v 3.6 (www.itksnap.org) by a radiologist with 7 years’ work experience. Next, the delineated intratumoral ROI was radially dilated (4 mm distance) outside the tumor using Python v3.6. Finally, the intratumoral ROI was subtracted from the dilated ROI to obtain the peritumoral ROI of the breast cancer. The delineated intratumoral ROI and dilated peritumoral ROI were used to calculate features from the intra- and peri-tumoral region, respectively. **Figure 2** shows an example of the ROI segmentation and dilation process for each modality in this study.

### Feature extraction

For each patient, a total of 15,178 radiomics features were extracted from intra- and peri-tumoral ROIs in the DM, DBT, DCE-MRI and DWI using the “Pyradiomics” package (19) in Python V3.6. The features were classified as first-order, shape-based and texture features. The texture features can be categorized into the gray level cooccurrence matrix (GLCM), gray level run length matrix (GLRLM), gray level size zone matrix (GLSZM), neighboring gray tone difference matrix (NGTDM), and gray level dependence matrix (GLDM). The high-dimensional features were also calculated from the transformed images that were filtered with eight types of filters (Wavelet, Laplacian of Gaussian, Square, Squareroot, Logarithm, Exponential, Gradient and Localbinarypattern) based on the first-order and texture features.

### Feature selection

The Mann-Whitney U test was performed on the extracted features. Features with P < 0.05 were considered significantly variables between the SLN- and SLN+ groups, and remained. Next, the least absolute shrinkage and selection operator (LASSO) with 10-fold cross-validation was employed to select the most significant features suggestive of SLN status. Finally, the logistic regression with Akaike Information Criterion (AIC) (20) as the stopping rule was used to identify the most predictive features.

### Radiomics signature development and validation

The radiomics signature was developed by linearly fitting the predictive features weighted by the corresponding LASSO coefficients. The ROC curve was drawn to evaluate the prediction performance of the radiomics signature using the “sklearn” package in R v.3.6. The Delong’s test was used to compare different ROC curves. Area under the ROC curve (AUC), accuracy (ACC), specificity (SPE), sensitivity (SEN) and negative predictive value (NPV) were used as comparison metrics.

## Results

### Patient characteristics

A total of 208 invasive breast cancer patients (51.98 ± 9.99 years; mean age, 51.98 year) were finally included, among which 110 (53%) were SLN- and 98 (47%) were SLN+. **Table 1** listed all patients and their clinical characteristics. Statistical analysis showed that the Ki-67 status was significant different between the SLN- and SLN+ groups with *P*<0.05. No significant difference was found between the two groups in regard to age, menstruation status, tumor location, histological grade, histological type, ER status, PR status and HER-2 status (*P*>0.05).

**Table 1:** Patients and clinical characteristics.

Characteristic	SLN- group (n = 110)	SLN+ group (n = 98)	*P* value
Age (Mean ± SD)	51.95 ± 10.19	52.01 ± 9.80	0.660
Menstruation status			0.150
Premenopausal	57 (51.8%)	41 (41.8%)	
Postmenopausal	53 (48.2%)	57 (58.2%)	
Tumor location			0.255
UIQ	20 (18.2%)	12 (12.2%)	
UOQ	56 (50.9%)	42 (42.9%)	
LOQ	2 (1.8%)	5 (5.1%)	
LIQ	10 (9.1%)	13 (13.3%)	
Central	22 (20.0%)	26 (26.5%)	
Histological grade			0.094
I	4 (3.6%)	0 (0.0%)	
II	85 (77.3%)	84 (85.7%)	
III	21 (19.1%)	14 (14.3%)	
Histological type			0.102
IDC	110 (100.0%)	94 (95.9%)	
Other	0 (0.0%)	4 (4.1%)	
ER status			0.873
-	27 (24.5%)	25 (25.5%)	
+	83 (75.5%)	73 (74.5%)	
PR status			0.180
-	35 (31.8%)	23 (23.5%)	
+	75 (68.2%)	75 (76.5%)	
HER-2 status			0.148
-	85 (77.3%)	67 (60.9%)	
+	25 (22.7%)	31 (31.6%)	
Ki-67 level			0.006*
Low	29 (26.4%)	11 (11.2%)	
High	81 (73.6%)	87 (88.8%)	

SLN-, sentinel lymph node negative; SLN+, sentinel lymph node positive; SD, standard deviation; UIQ, upper inner quadrant; UOQ, upper outer quadrant; LOQ, lower outer quadrant; LIQ, lower inner quadrant; IDC, invasive ductal carcinoma; ER, estrogen receptor; PR, progesterone receptor; HER-2, human epidermal growth factor receptor-2. * p < 0.05.

### Prediction performance of intratumoral regions, peritumoral regions, and their combination

**Figure 3:**
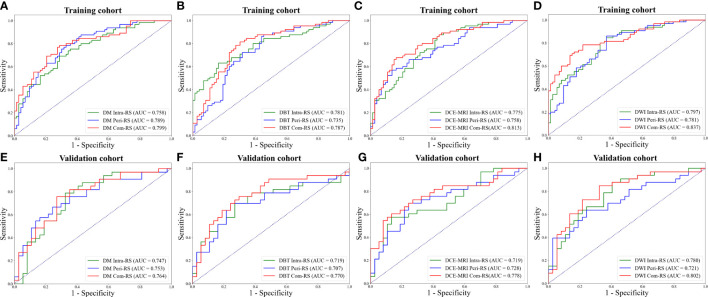
ROC curves of each developed RS. **(A, E)** ROC curves for the RSs from DM. **(B, F)** ROC curves for the RSs from DBT. **(C, G)** ROC curves for the RSs from DCE-MRI. **(D, H)** ROC curves for the RSs from DWI. The above row represents ROC curves in the training cohort, whereas the bottom row represents ROC curves in the validation cohort.

**Table 2** demonstrated performance of the radiomics signatures derived from intra- (Intra-RS) and peri-tumoral regions (Peri-RS), and combination of intra- and peri-tumoral regions (Com-RS) based on DM, DBT, DCE-MRI and DWI separately. **Figure 3** showed ROC curves of each developed RS. For DM and DCE-MRI, the Intra-RS and Peri-RS produced similar predictive performance in terms of AUCs. For DBT and DWI, the Intra-RSs always generated higher AUCs than the Peri-RSs. For each modality, the Com-RSs always yielded higher AUCs compared with the Intra-RSs and Peri-RSs, which indicates that the intra- and peri-tumoral regions may hold complementary information. The Com-RS derived from the DWI was superior to those derived from other modalities in terms of AUC, ACC, SEN and NPV. While, the Com-RS derived from the DCE-MRI outperforms those derived from other modalities in regard to SPE.

**Table 2:** Prediction performance of RSs derived from intra-, peri- and combined tumoral regions.

Modality	Model	Training cohort					Validation cohort				
		AUC	ACC	SPE	SEN	NPV	AUC	ACC	SPE	SEN	NPV
DM	Intra-RS	0.758	0.703	0.712	0.692	0.722	0.750	0.729	0.676	0.788	0.781
	Peri-RS	0.789	0.725	0.671	0.785	0.778	0.753	0.714	0.757	0.667	0.718
	Com-RS	0.798	0.746	0.712	0.785	0.788	0.764	0.743	0.730	0.758	0.771
DBT	Intra-RS	0.781	0.739	0.836	0.631	0.718	0.719	0.714	0.730	0.697	0.730
	Peri-RS	0.735	0.703	0.548	0.877	0.833	0.707	0.729	0.757	0.697	0.737
	Com-RS	0.787	0.754	0.699	0.815	0.810	0.770	0.729	0.703	0.758	0.765
DCE-MRI	Intra-RS	0.775	0.710	0.562	0.877	0.837	0.719	0.729	0.865	0.576	0.696
	Peri-RS	0.758	0.725	0.863	0.569	0.692	0.728	0.729	0.757	0.697	0.737
	Com-RS	0.813	0.761	0.836	0.677	0.744	0.778	0.743	0.865	0.606	0.711
DWI	Intra-RS	0.797	0.732	0.630	0.846	0.821	0.780	0.729	0.595	0.879	0.846
	Peri-RS	0.781	0.739	0.630	0.862	0.836	0.721	0.700	0.757	0.636	0.700
	Com-RS	0.837	0.783	0.781	0.785	0.803	0.802	0.757	0.676	0.848	0.833

AUC, area under the receiver operating characteristic curve; ACC, accuracy; SEN, sensitivity; SPE, specificity; NPV, negative predictive value.

### Comparisons of prediction performance of mammography and MRI

**Figure 4:**
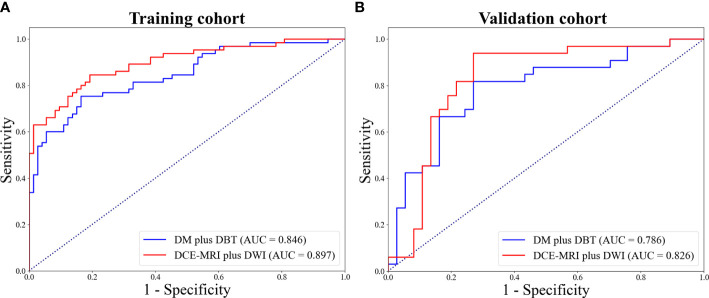
ROC curves for DW plus DBT and DCE-MRI plus DWI in the training **(A)** and validation **(B)** cohort.

The Predictive performance of mammography (DW plus DBT) and MRI (DCE-MRI plus DWI) techniques were compared and listed in **Table 3**. The DCE-MRI plus DWI exhibited better performance compared to DM plus DBT in terms of AUC, ACC, SEN and NPV. **Figure 4** showed ROC curves of the models. The prediction performance of DM plus DBT generated better AUC, ACC, SPE and NPV than DM or DBT alone (compare **Table 3** with **Table 2**). The DCE-MRI plus DWI outperformed DCE-MRI or DWI alone in regards to AUC, ACC, SEN and NPV.

**Table:** Table 3 Comparisons of prediction performance of mammography and MRI.

Model	Training cohort						Validation cohort					
	AUC	ACC	SPE	SEN	NPV	*P*	AUC	ACC	SPE	SEN	NPV	*P*
M1	0.846	0.797	0.836	0.754	0.792		0.786	0.771	0.730	0.818	0.818	
M2	0.897	0.826	0.808	0.846	0.855		0.826	0.829	0.730	0.939	0.931	
M1 vs. M2						0.239						0.591

M1, DM plus DBT; M2, DCE-MRI plus DWI.

Detailed information of the selected features from mammography and MRI are listed in **Table 4**. For the mammography, a total of 5 and 5 features were ultimately selected from the intra- and peri-tumoral regions, respectively. For the MRI, a total of 8 and 3 features were selected from the intra- and peri-tumoral regions, respectively. All features belong to high-dimensional feature classes. P values of the selected features were all less than 0.05 in the training cohort.

**Table 4:** The selected most important features from mammography and MRI.

Feature	Source	Region	Cohort	Mean ± SD		AUC	P
				SLN -	SLN +		
**Mammography**							
wavelet-HHH_glcm_ClusterShade	DM	Intra	Training	0.0003 ± 0.003	-0.0008 ± 0.001	0.656	0.002
			Validation	0.0050 ± 0.032	-0.0009 ± 0.002	0.615	0.098
wavelet-LHL_firstorder_Skewness	DM	Intra	Training	-0.060 ± 0.209	0.017 ± 0.139	0.647	0.003
			Validation	-0.013 ± 0.057	-0.006 ± 0.054	0.603	0.140
exponential_firstorder_Maximum	DM	Peri	Training	143.912 ± 104.405	215.192 ± 131.260	0.673	<0.001
			Validation	132.973 ± 85.730	201.302 ± 85.303	0.716	0.002
lbp-3D-m2_glrlm_RunVariance	DM	Peri	Training	2099.409 ± 803.178	2428.540 ± 690.629	0.602	0.039
			Validation	2290.893 ± 616.195	2705.752 ± 1209.431	0.622	0.079
wavelet-HHH_firstorder_Mean	DM	Peri	Training	7.196E-19 ± 3.459E-18	-6.645E-19 ± 3.193E-18	0.613	0.016
			Validation	-5.921E-20 ± 4.791E-18	-1.040E-18 ± 2.720E-18	0.546	0.290
wavelet-LHH_firstorder_Mean	DM	Peri	Training	5.512E-19 ± 3.641E-18	2.186E-18 ± 4.543E-18	0.602	0.022
			Validation	1.035E-18 ± 4.015E-18	1.579E-18 ± 2.063E-18	0.566	0.473
lbp-2D_glszm_SmallAreaEmphasis	DBT	Intra	Training	0.436 ± 0.095	0.483 ± 0.105	0.648	0.003
			Validation	0.426 ± 0.089	0.490 ± 0.127	0.620	0.085
logarithm_glszm_LargeAreaLowGrayLevelEmphasis	DBT	Intra	Training	4252.645 ± 3581.690	8644.131 ± 10956.919	0.604	0.035
			Validation	4424.162 ± 7175.345	5172.355 ± 4966.965	0.602	0.143
logarithm_glszm_ZonePercentage	DBT	Intra	Training	0.028 ± 0.011	0.024 ± 0.009	0.624	0.012
			Validation	0.028 ± 0.007	0.025 ± 0.007	0.658	0.024
exponential_glcm_MaximumProbability	DBT	Peri	Training	0.486 ± 0.202	0.402 ± 0.159	0.623	0.013
			Validation	0.431 ± 0.171	0.361 ± 0.167	0.613	0.103
**MRI**							
lbp-2D_glcm_Imc1	DCE-MRI	Intra	Training	-0.021 ± 0.011	-0.017 ± 0.009	0.601	0.041
			Validation	-0.024 ± 0.009	-0.018 ± 0.006	0.695	0.005
lbp-3D-m2_glszm_GrayLevelVariance	DCE-MRI	Intra	Training	1.293 ± 0.267	1.184 ± 0.266	0.623	0.013
			Validation	1.269 ± 0.335	1.199 ± 0.245	0.565	0.350
wavelet-HLL_firstorder_Mean	DCE-MRI	Peri	Training	1.897 ± 1.385	2.430 ± 1.296	0.613	0.022
			Validation	1.768 ± 1.484	2.773 ± 1.307	0.710	0.003
exponential_glrlm_RunEntropy	DWI	Intra	Training	3.728 ± 1.075	3.352 ± 1.139	0.605	0.034
			Validation	3.297 ± 1.112	3.523 ± 0.971	0.565	0.350
lbp-2D_gldm_LargeDependenceHighGrayLevelEmphasis	DWI	Intra	Training	199.140 ± 84.247	170.020 ± 72.939	0.605	0.034
			Validation	188.375 ± 102.102	185.655 ± 71.711	0.516	0.819
lbp-3D-k_glrlm_LowGrayLevelRunEmphasis	DWI	Intra	Training	0.896 ± 0.083	0.928 ± 0.052	0.612	0.024
			Validation	0.895 ± 0.083	0.925 ± 0.054	0.596	0.169
lbp-3D-m1_glszm_SizeZoneNonUniformityNormalized	DWI	Intra	Training	0.197 ± 0.061	0.185 ± 0.071	0.598	0.047
			Validation	0.187 ± 0.054	0.178 ± 0.059	0.563	0.362
lbp-3D-m2_glcm_MCC	DWI	Intra	Training	0.611 ± 0.168	0.546 ± 0.117	0.617	0.018
			Validation	0.616 ± 0.161	0.538 ± 0.114	0.650	0.031
wavelet-HHH_firstorder_Skewness	DWI	Intra	Training	-0.043 ± 0.105	0.013 ± 0.147	0.618	0.017
			Validation	-0.041 ± 0.165	0.021 ± 0.135	0.564	0.356
log-sigma-1-0-mm-3D_glszm_ZoneVariance	DWI	Peri	Training	1.046 ± 0.933	2.514 ± 10.897	0.651	0.002
			Validation	1.514 ± 2.009	0.746 ± 0.543	0.599	0.156
wavelet-HLH_gldm_SmallDependenceHighGrayLevelEmphasis	DWI	Peri	Training	999.432 ± 748.358	1587.385 ± 1529.790	0.600	0.042
			Validation	875.486 ± 853.221	1593.934 ± 1194.784	0.704	0.003

SD, standard deviation.

## Discussion

To the best of our knowledge, this is the first attempt to comprehensively investigate values of DM, DBT, DCE-MRI and DWI individually and in combination for prediction of the SLN status in BC. Previous studies on predicting the SLN status focused solely on the intratumoral region of BC (2, 21–23), which ignored information from tissues surrounding the tumor. We found that for each modality, the Intra-RS and Peri-RS have comparable predictive capabilities, yielding similar AUC and accuracy. By combing the intra- and peri-tumoral regions, the Com-RS can always efficiently improve the predictive performance compared with the Intra-RS or Peri-RS alone, in the four modalities. Therefore, our results indicated that the intra- and peri-tumoral regions may hold complementary information on the SLN status. This was partially consistent with some recent reports that demonstrated values of peritumoral regions for the assessment of benign and malignant patterns (24), prediction of HER2-enriched (25) and pretreatment evaluation of responses to neoadjuvant chemotherapy (nCRT) (26) in patients with breast tumor. Our study indicated that the peritumoral region was also highly correlated with the SLN status and should be paid attentions to in future researches.

In addition, most prior studies related to our work only assessed a single modality [e.g., DCE-MRI (21, 22)] for predicting the SLNM, which is inherently limited. Besides, the MRI has a high examination fee and is not suitable for all patients (9). The mammography, on the other hand, is accessible for most patients. While, mammography-based radiomics prediction of the SLNM in BC has not been investigated. By comparing performance of mammography (DM plus DBT) and MRI (DCE-MRI plus DWI) in a direct and quantitative manner, we found that the DCE-MRI plus DWI generated higher AUC, accuracy, sensitivity and negative predictive value compared with DM plus DBT. It is explainable considering the fact that breast lesions are frequently obscured in mammography imaging due to the overlap between the lesion site and glandular tissue (27). This was partially in line with recent studies that found that breast mammography produced lower AUC and sensitivity than MRI for the diagnosis of BC (28, 29). While, we found that the DM plus DBT showed slightly increased specificity compared with DCE-MRI plus DWI, which was in accordance with previous reports that demonstrated relatively low specificity of MRI in the diagnosis of BC (9). Our study indicated that the MRI may result in higher rates of misdiagnosis on SLNM compared with mammography.

For each modality, we comprehensively analyzed radiomics features from both the tumor and tissues surrounding the tumor, and finally identified 11 and 10 features from mammography and MRI, respectively. For MRI, most of the features (8 of 11) were derived from the intratumoral region, the majority of which (7 of 8 features) belong to the textural feature class. The textural features are based on statistics and can provide great amount of detail regarding intratumoral heterogeneity (30). Therefore, our finding may suggest that the MRI may be better in capturing heterogeneity characteristics within the tumor compared to mammography. All identified features are filtered features, and thus cannot be understood by radiologists, which may explain why the SLN status can hardly be assessed through visual examinations.

There are limitations in our study. First, this is a retrospective investigation based on a single center, which may introduce bias. Second, the study only evaluated handcrafted radiomics features. Deep learning-based features should be assessed in our future work. Third, this study analyzed peritumoral regions and the whole tumor region, but not comprehensively investigated the intratumoral heterogeneity distribution. Further studies should explore values of subregional radiomics for predicting the SLNM, other than extracting features across the entire tumor mass.

## Conclusion

In conclusion, radiomics features from intra- and peri-tumoral regions can provide complementary information to identify the SLNM. The DCE-MRI plus DWI generated lower specificity, but higher AUC, ACC, sensitivity and negative predictive value compared with DM plus DBT. Our findings may contribute to a better knowledge of intra- and peri-tumoral regions in various modalities for prediction of SLNM in BC.

## Data availability statement

The data and material that support the findings of this study are available from the corresponding author upon reasonable request. Requests to access these datasets should be directed to nanazhao888@outlook.com.

## Ethics statement

The studies involving human participants were reviewed and approved by The ethics committee of Liaoning Cancer Hospital and Institute. Written informed consent for participation was not required for this study in accordance with the national legislation and the institutional requirements.

## Author contributions

YC, SX, and HW: study design. YC, SX, and HW: data collection. YC, XW, SN, and YL: data analysis and interpretation. YC and NZ: manuscript writing. NZ: funding acquisition. All authors contributed to the article and approved the submitted version.
